# Antiviral Activity of Two Marine Carotenoids against SARS-CoV-2 Virus Entry In Silico and In Vitro

**DOI:** 10.3390/ijms22126481

**Published:** 2021-06-17

**Authors:** Sung-Kun Yim, Inhee Kim, Boyd Warren, Jungwon Kim, Kyoojin Jung, Bosung Ku

**Affiliations:** 1 Marine Biotechnology Research Center, Jeonnam Bioindustry Foundation, 21-7, Nonggongdanji 4Gil, Wando-eup, Wando-gun, Jeollanam-do 59108, Korea; boydtwarren@gmail.com (B.W.); kjjung@jbf.kr (K.J.); 2 Medical & Bio Decision (MBD) Co. Ltd., #B-8F, 145 Gwanggyo-ro, Yeongtong-gu, Suwon-si, Gyeonggi-do 16229, Korea; ihkim@mbdbiotech.com (I.K.); jungwonk@mbdbiotech.com (J.K.); goos4684@mbdbiotech.com (B.K.)

**Keywords:** COVID-19, siphonaxanthin, fucoxanthin, carotenoid, seaweed, antiviral activity, molecular docking

## Abstract

The marine carotenoids fucoxanthin and siphonaxanthin are powerful antioxidants that are attracting focused attention to identify a variety of health benefits and industry applications. In this study, the binding energy of these carotenoids with the SARS-CoV-2 Spike-glycoprotein was predicted by molecular docking simulation, and their inhibitory activity was confirmed with SARS-CoV-2 pseudovirus on HEK293 cells overexpressing angiotensin-converting enzyme 2 (ACE2). Siphonaxanthin from *Codium fragile* showed significant antiviral activity with an IC50 of 87.4 μM against SARS-CoV-2 pseudovirus entry, while fucoxanthin from *Undaria pinnatifida* sporophyll did not. The acute toxicities were predicted to be relatively low, and pharmacokinetic predictions indicate GI absorption. Although further studies are needed to elucidate the inhibition of viral infection by siphonaxanthin, these results provide useful information in the application of these marine carotenoids for the treatment and prevention of COVID-19.

## 1. Introduction

Since the first approval of a COVID-19 vaccine in December 2020, more than 1.04 billion doses have been administered in 172 countries [1,2,3], but COVID-19 is still spreading worldwide with multiple variants [4] and causing tremendous fear. According to Bloomberg, the latest vaccination rate is an average of 19,214,353 doses per day worldwide, and they estimate that it will take another 18 months to cover 75% of the population with a two-dose vaccine at the current rate [3,4]. Although vaccination has begun worldwide, it will take a long time to safely achieve herd immunity against COVID-19 by vaccine, so effective therapies are still needed to prevent or treat COVID-19 [3].

Seaweeds are a rich source of carotenoids [5] such as fucoxanthin, lutein, β-carotene, and siphonaxanthin. Such carotenoids are powerful antioxidants that can protect the body from oxidative stress and have been extensively studied for the prevention of cardiovascular diseases, neurodegenerative diseases, and cancer [6,7]. One of the polar xanthophylls, fucoxanthin (FX), which is a characteristic orange, is abundant in several brown seaweeds including *Undaria pinnatifida* [8,9], *Hijikia fusiformis* [10], *Laminaria japonica* [11], *Sargassum* sp. [12,13,14,15,16], and *Fucus* sp. [17]. It is one of the most abundant carotenoids in seaweeds, accounting for more than 10% of the estimated total natural production of carotenoids [18]. Another polar xanthophyll, siphonaxanthin (SX), is a xanthophyll found in green algae such as *Caulerpa lentillifera*, *Codium fragile*, and *Codium cylindricum* [19]. As an oxidative metabolite of lutein, it possesses a structure similar to lutein except for one keto group located at C-8 and an extra hydroxyl group at C-19. These carotenoids have been shown to possess several potential bioactivities such as anticancer, anti-inflammatory, anti-obesity, and neuroprotective effects [20,21,22]. Naturally sourced carotenoids may be an underexplored source for novel antiviral treatments.

In this study, we investigated the inhibitory activity of two polar xanthophylls, fucoxanthin from *Undaria pinnatifida* sporophyll and siphonaxanthin from *Codium fragile*, against SARS-CoV-2 virus infection (using the SARS-CoV-2 pseudovirus) in HEK293/ ACE2 cells. This study aims to provide useful information for the application of marine carotenoids beneficial to human health.

## 2. Results

### 2.1. Molecular Docking

**Figure 1:**
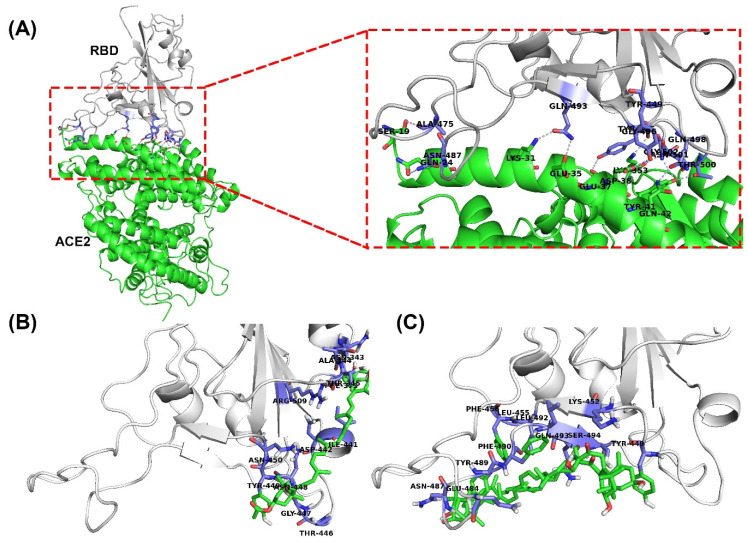
Surface and cartoon representations of structures of (**A**) the SARS-CoV-2 chimeric receptor-binding domain (RBD) complexed with angiotensin-converting enzyme 2 (ACE2). The molecular docking poses of (**B**) fucoxanthin and (**C**) siphonaxanthin interacting with RBD (PDB:6vw1) analyzed with AutoDock Vina. The tertiary structures of RBD-ligands were made with PyMOL. Ligands and ACE2 are depicted as green sticks and green cartoons, and the binding site residues are labeled accordingly.

**Figure 2:**
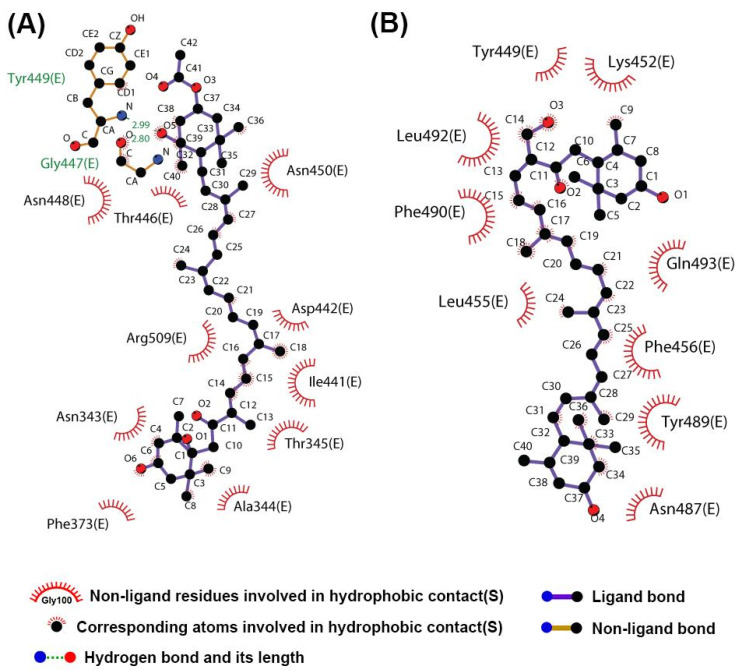
The potential protein–ligand interactions of SARS-CoV-2 chimeric receptor-binding domain (RBD) and ligands. The best-docked poses for (**A**) fucoxanthin and (**B**) siphonaxanthin in RBD were made with LigPlot.

After visual inspection of the top-ranked poses, the potential binding sites were found for the FX and SX models: the region corresponding to the binding site of the SARS-CoV-2 chimeric receptor-binding domain (RBD) with ACE2 (Figure 1) [23]. The binding energies and residue interactions with the test compounds are presented in Table 1. The binding energies of FX and SX for the RBD were −2.91 kcal/mol and −2.78 kcal/mol, respectively. FX displayed a higher binding energy than SX, but it could not bind at the RBD-ACE2 binding site (Figure 1A,B). On the other hand, SX could bind at that location (Figure 1A,C), but its binding energy was slightly lower than FX. As shown in Figure 2, the potential interaction of RBD with ligands was hydrophobic in nature, and the interacting residues between RBD and SX were similar to those of ACE2 (Tyr449, Tyr 489, and Gln 493), while FX bound to the surface of RBD (Figure 2A).

### 2.2. Cytotoxicity Assay

**Figure 3:**
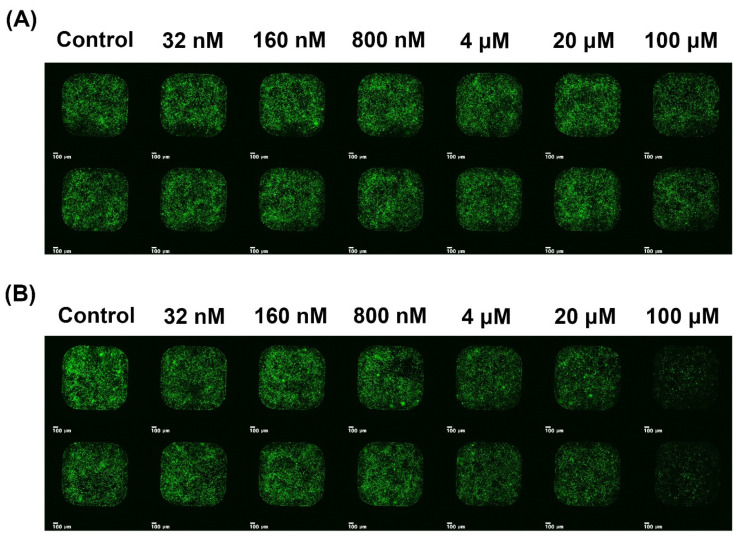
The GFP fluorescence, due to infection after treatment with pseudovirus and the indicated concentrations of (**A**) fucoxanthin and (**B**) siphonaxanthin for 96 h, in HEK293/ACE2 was scanned with an MBD ASFA scanner (MBD Biotech, Suwon, Korea). Green fluorescence indicates HEK293/ACE2 cells infected with pseudovirus. Scale bar: 100 μm.

The viability of HEK293/ACE2 cells was assessed using a Cell Titer-Glo Luminescent cell viability assay kit (Promega, Madison, WI, USA). When the HEK293/ACE2 cells were treated with FX and SX at the final concentration range from 32 nM to 100 μM (serially diluted 1/5) for 96 h, the test compounds did not show cytotoxicity until 100 μM, when they showed minor cytotoxicity (Figure 3).

### 2.3. Inhibition of Viral Infection

Inhibition of viral infection by FX and SX was performed with a SARS-CoV-2 pseudovirus. As shown in Figure 2 and Figure 3, SX inhibited SARS-CoV-2 pseudovirus infection of HEK293/ACE2 cells at various concentrations. After exposure, the viral infection rate was visualized by GFP fluorescence imaging, which is presented in Figure 3. It was observed that viral infection mostly decreased with the increasing concentrations of SX. The SX showed antiviral activity with an IC50 of 87.4 μM, while FX displayed no antiviral activity against SARS-CoV-2 entry.

### 2.4. Acute Toxicity and ADME Prediction

General Unrestricted Structure‒Activity Relationships (GUSAR) software [24] was used for quantitative in silico toxicity prediction for FX and SX in rats with four types of administration (oral, intravenous, intraperitoneal, and subcutaneous). As shown in Table 2, the difference in LD50 values obtained for the four different routes suggests that availability of the tested compound for metabolism by the liver is a major factor in its toxicity. The LD50 value of SX was higher than FX for intravenous (IV), oral, and subcutaneous (SC) routes of administration, but the LD50 value of FX was higher through intraperitoneal (IP) administration. By the OECD chemical classification system, FX and SX are Class 4 when administered through IP and oral routes, whereas they are Class 3 for IV and SC routes of administration. The ADME (absorption, distribution, metabolism, and elimination) properties of FX and SX were predicted by SwissADME [25] (Table 3). FX and SX had lipophilicities of 6.78 and 6.53 (Log Po/w), respectively, and low GI adsorption. Moreover, their bioavailability scores and water solubilities were 0.17 and poorly soluble, respectively.

## 3. Discussion

Two polar xanthophylls, FX from *Undaria pinnatifida* sporophyll and SX from *Codium fragile*, have been shown to possess several potential bioactivities including anticancer, anti-inflammatory, and anti-obesity effects, and serve as powerful antioxidants [20,21,22]. In this present study, whether these compounds can bind in the ACE2 binding region of the SARS-CoV-2 spike-protein was predicted using AutoDockTools and the inhibition of SARS-CoV-2 entry by these compounds was confirmed using a SARS-CoV-2 pseudovirus and HEK293/ACE2 in vitro.

In molecular docking, FX and SX displayed low binding energy (−2.91 and −2.78 kcal/mol, respectively), but SX fit exactly into the ACE2 binding region of RBD (Figure 1C), unlike FX (Figure 1B). The reason seems to be that FX is more hydrophobic than SX. Fucoxanthin has an epoxide and an allenic bond in its structure, whereas siphonaxanthin does not contain either of those functional groups. However, it possesses an additional hydroxyl group on the 19th carbon atom [26]. In SwissADME results, the lipophilicity of FX is higher (6.78) than SX (6.53), and the water solubility of FX is lower (−8.2) than SX (−8.0) in silico (Table 3). These results support the conclusion that SX is more hydrophilic than FX and could more easily bind to the binding site of S-glycoprotein for ACE2. As in Unni et al. [27], the binding site was divided into three hydrophilic regions based on the electrostatic character of the surface in each region of the S-glycoprotein–ACE2 interface, referred to as Site1 (Gly446, Tyr449, Gly496, Gln498, Thr500, and Asn501), Site 2 (Lys417 and Gln493), and Site 3 (Ala475 and Asn487) on the S-glycoprotein. The interaction of SX and S-protein spanned Site 1 (Tyr449), Site2 (Gln493), and Site 3 (Asn487), but FX only made contact with Site 1 (Thr446 and Tyr449).

**Figure 4:**
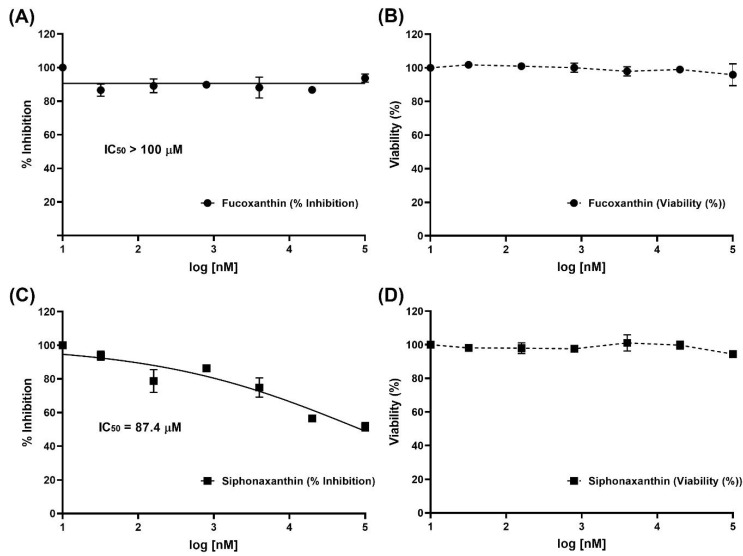
Determination of the cytotoxicity and antiviral activity of the fucoxanthin and siphonaxanthin in HEK293/ACE2 cells. The inhibition of viral infection by the (**A**) fucoxanthin and (**C**) siphonaxanthin was performed with SARS-CoV-2 pseudovirus containing the S-glycoprotein of SARS-CoV-2 (COVID-19). The viability of HEK293/ACE2 cells was assessed using an CellTiter-Glo^®^ Luminescent cell viability assay kit (Promega, Madison, WI, USA) after treatment with the indicated concentrations of the (**B**) fucoxanthin and (**D**) siphonaxanthin for 96 h. Results are expressed as percent of inhibition in drug-treated cultures compared with untreated and was plotted with GraphPad Prism software (Graph-Pad, San Diego, CA, USA). Values are the means ± S.D. (*n* = 3).

In vitro, the antiviral activities of SX and FX against SARS-CoV-2 fit our expectations from the molecular docking simulations. FX displayed no antiviral activity (Figure 4A), while SX showed antiviral activity with an IC50 of 87.4 μM (Figure 4C) against SARS-CoV-2 entry. These results indicate that SX effectively interferes with interaction of S-glycoprotein and ACE2, but FX does not. In recent studies, sulfated polysaccharides [28,29], fucoidan [30], and some crude polysaccharide extractions [31] effectively inhibited SARS-CoV-2 entry in vitro. According to studies so far, highly branched and sulfated polysaccharides with high molecular weight can effectively inhibit COVID-19 viral infection. Although carotenoids are known to have beneficial effects on health and diverse applications [32], such as antibacterial, antiviral, antifungal, anticancer, antidiabetic, anti-inflammatory, antioxidant, anti-obesity, and neuroprotective applications, there are no studies related to how marine carotenoids can inhibit SARS-CoV-2 entry. These results show for the first time that SX, a marine carotenoid rather than polysaccharide, significantly inhibits SARS-CoV-2 viral infection without cytotoxicity at a dose of less than 100 μM.

Determining the acute toxicity and pharmacokinetic properties of these marine carotenoids was facilitated by GUSAR software [24] and SwissADME [25]. The toxicity profile of these compounds is relatively low, and they require high doses to elicit toxic responses. As shown in Table 2, FX and SX are Class 4 chemicals that have mild toxic effects when administered orally or intraperitoneally, but they are Class 3 intravenously and subcutaneously. Commercially, FX and SX are sold as health supplements because these compounds are believed to possess a variety of health benefits [20,21,22]. However, in previous studies, FX inhibited the activity of CYP1A2 and CYP3A4 with an IC50 of 30.3 μM and 24.4 μM, respectively [33,34,35]. These CYPs are responsible for the metabolism of more than 60% of commonly prescribed drugs [36]. The inhibition by FX against CYPs may reduce the effects of drugs that are administered as a pro-drug which needs to be broken down into its active form, and it may also enhance the cytotoxicity of drugs by causing them to linger in the body longer, and at higher levels than expected. SX did not show the inhibition of CYP1A2 and CYP3A4 activities in vitro, but little information on its in vitro cytotoxicity and in vivo bioavailability and biotransformation is available. Recently, Li et al. [37] reported that SX mainly accumulates in the stomach and small intestine but was detected in the plasma and most tissues (except the bladder) of mice at the end of a 16-day dietary supplementation with SX (0.004%), while putative metabolites of SX mainly accumulated in liver and adipose tissues. These observations suggest that SX can be absorbed and metabolized in mice, but the ultimate destiny of SX in the human body requires further study.

In summary, SX inhibits SARS-CoV-2 virus infection even in vitro, as predicted by molecular docking in silico. Additionally, SX has been shown to possess several potential bioactivities including anticancer, anti-inflammatory, and anti-obesity effects, and it is a powerful antioxidant. Although further studies are needed to elucidate the inhibition mechanisms of SX against SARS-CoV-2 virus infection and identify the biological activities of SX in vitro and vivo when SARS-CoV-2 virus is present, this study provides useful information for application of these beneficial marine carotenoids in improving human health.

## 4. Materials and Methods

### 4.1. Chemicals and Reagents

All analytical grade organic solvents including hexane, chloroform, acetonitrile, and methanol were purchased from Burdick & Jackson chemicals (Muskegon, MI, USA). Ultra-pure argon (99.99%), nitrogen (99.99%), and carbon dioxide were supplied from Daechang Gas (Songha-dong, Gwangju, South Korea). Fucoxanthin (FX) and siphonaxanthin (SX) were extracted and purified from sporophyll of *Undaria pinnatifida* and *Codium fragile*, respectively, for a previous study [35].

### 4.2. Docking Studies

Chemical structures of FX (Compound CID:5281239) and SX (Compound CID:11204185) were obtained from PubChem database (https://pubchem.ncbi.nlm.nih.gov). Test compounds in sdf format were formatted to pdbqt files with OpenBabel [38]. The three-dimensional structures for SARS-CoV-2 chimeric RBD (PDB: 6VW1, chain C) [23] was downloaded from the Protein Data Bank (https://www.rcsb.org) [39]. The removal of counter-ions, crystallographic waters, and other ligands (except the heme group) and the addition of atomic charges and solvation parameters were done using AutoDockTools (version 1.5.6) [40]. The ligand ACE2 (angiotensin-converting enzyme 2, PDB: 6VW1, chain A) was used as the control for the SARS-CoV-2 spike-glycoprotein. Docking calculations were carried out using AutoDock Vina (version 1.1.2) [41]. Grids were centered on coordinates 63.141, −13.492, and 185.392 with 1.0 Å grid spacing and dimensions of 60 Å × 60 Å × 60 Å on *x-*, *y-*, and *z*-axes for the RBD. The top-ranked binding modes and protein–ligand interactions were visualized with PyMOL Molecular Graphics system (Shrödinger, LLC, version 1.8), Protein–ligand Interaction Profiler [42], and LigPlot [43].

### 4.3. SARS-CoV-2 Pseudovirus and Cell

SARS-CoV-2 Pseudovirus (COV-PS02) expressing S-glycoprotein on the surface of the Lentivirus and HEK293/ACE2 cell genetically engineered to overexpress angiotensin-converting enzyme 2 were purchased from Creative Diagnostics (Shirley, NY, USA). Dulbecco’s modified Eagle’s medium (DMEM), fetal bovine serum (FBS), and geneticin (G-418 sulfate) were purchased from Thermo Fisher Scientific (Waltham, MA, USA), and the Cell Titer-Glo Luminescent cell viability assay kit was purchased from Promega (Madison, WI, USA). HEK293/ACE2 was maintained at 37 °C, 5% CO_2_ in Dulbecco’s modified Eagle medium (DMEM) supplemented with 10% (*v*/*v*) heat-inactivated fetal bovine serum and 0.5 mg mL^−1^ of G418. The cells were sub-cultured within 48 h intervals.

### 4.4. Cytotoxicity Assay

The cytotoxicity evaluation of FX and SX was performed using a Cell Titer-Glo Luminescent cell viability assay kit. After the HEK293/ACE2 cells were each treated with 20 μL of FX or SX at various concentrations (5-fold serial dilutions in the range of 32 nM to 100 μM, final DMSO 1%) for 96 h, 4 μL/well of Cell titer Glo reagent was added, and the plate was shaken for 2 min at 700 rpm. After 10 min, the mixture was read via luminometer and cell viability was calculated as follows:

(1) $$Cell\;Viability\;\left(\%\right)=\frac{RLU\;sample}{RLU\;conc.}\times 100$$

where the *RLU* sample is the luminescence of the experimental sample, and *RLU* conc. is the luminescence of the control. Cytotoxicity was calculated as follows:

(2) Cytotoxicity (%)=100−% Cell Viability

The 50% cytotoxic concentration for FX and SX was determined using nonlinear regression analysis with GraphPad Prism software (Graph-Pad, San Diego, CA, USA). Each sample was analyzed in duplicate wells and repeated three times.

### 4.5. Inhibition of Viral Infection

To investigate the inhibitory effects of FX and SX on viral infection, HEK293/ACE2 was seeded in 384-well plates at a concentration of 2 × 10^3^ cells/well using DMEM and incubated at 37 ˚C, 5% CO_2_ for 24 h. When the HEK293/ACE2 cells had grown to a density of 30–40% in a 384-well plate, the cells were treated with the mixture of each 20 μL of FX or SX (serial diluted 1/5 in a concentration range of 32 nM to 100 μM, final DMSO 1%) and 1 μL of SARS-CoV-2 pseudovirus (titer: 1.0 × 10^7^ TU mL^−1^) to induce viral infection. After 96 h incubation, the viral infection rate was analyzed by scanning for GFP fluorescence with an MBD ASFA scanner (MBD Biotech., Suwon, South Korea). To calculate the inhibition of cell penetration by the SARS-CoV-2 pseudovirus, the GFP fluorescence area of infected cells was analyzed with an MBD cell analyzer and was calculated as follows:

(3) $$\%\;inhibition=\frac{\left(GFP\;AREA\;sample\right)}{\left(GFP\;AREA\;conc.\right)}\times 100$$

where the *GFP AREA* sample is the GFP area (μm^2^) of the experimental sample and *GFP AREA* conc. is the *GFP* area (μm^2^) of the control. Antiviral activity was calculated as follows:

(4) Antiviral activity (%)=100−% inhibition

Each sample was analyzed in triplicate, and the plots were made with GraphPad Prism software (Graph-Pad, San Diego, CA, USA).

### 4.6. ADMET Prediction

FX and SX were investigated for ADMET (adsorption, distribution, metabolism, excretion, and toxicity) properties in silico. The acute toxicity in rodent models and chemical classification of the test compounds were predicted by GUSAR [24]. It analyzes compounds based on the Quantitative Neighborhoods of Atoms descriptors and Prediction of Activity Spectra for Substances algorithm and correlates the obtained results with the SYMYX MDL toxicity Database and further classifies them based on the Organisation for Economic Co-operation and Development (OECD) chemical classification manual. The pharmacokinetics parameters were predicted by SwissADME [25] to ascertain the behavior of these compounds inside an organism in terms of absorption, distribution, metabolism, and excretion.

### 4.7. Statistical Analysis

The area of fluorescent cells infected by the pseudovirus was measured using the MBD Cell Analyzer. All experiments were performed in duplicate wells and repeated three times. The half maximal inhibitory concentrations (IC_50_) were calculated in GraphPad Prism 9 software using nonlinear regression analysis with log (inhibitor) plotted against the normalized response (variable slope). The equation corresponds to:

(5) Y=100/(1+10^((logIC50−X)×HillSlope))

where *Y* is the response, *X* is the logarithm of doses or concentrations, and HillSlope describes the steepness of the family of curves.

## Figures and Tables

**Table 1:** Interaction and binding energy of ligands with SARS-CoV-2 chimeric receptor-binding domain (RBD) in silico.

Compounds	SARS-CoV-2 Chimeric Receptor-Binding Domain (PDB:6VW1)	
	Binding Energy (kcal mol^−1^)	Interactions
ACE2	n.d.	Tyr449, Ala475, Asn487, Tyr489, Gln493, Gly496, Gln498, Thr500, Asn501, Gly502, Tyr505,
Fucoxanthin	−2.91	Asn343, Ala344, Thr345, Phe373, Ile441, Asp442, Thr446, Gly447, Asn448, Tyr449, Asn450, Arg509
Siphonaxanthin	−2.78	Tyr449, Lys452, Leu455, Phe456, Glu484, Asn487, Tyr489, Phe490, Leu492, Gln493, Ser494

ACE2: angiotensin-converting enzyme 2; n.d.: no data.

**Table 2:** Prediction of acute toxicity in silico by GUSAR in rodent models and chemical classification of compounds.

Ligands	Rat Oral LD50 (mg/kg)	Rat IV LD50 (mg/kg)	Rat SC LD50 (mg/kg)	Rat IP LD50 (mg/kg)	OECD Chemical Classification
Fucoxanthin	1,752,000	14,250	23,590	453,800	Class 3 and 4
Siphonaxanthin	1,907,000	17,070	52,120	434,400	Class 3 and 4

IP: Intraperitoneal; IV: Intravenous; LD50: Lethal dosage-50; OECD: Organisation for economic co-operation and development; SC: Subcutaneous.

**Table 3:** Pharmacokinetic parameters of compounds by SwissADME in silico.

Ligands	Log Po/w (Lipophilicity)	GI Absorption	BBB Permeant	CYP1A2 Inhibitor	Druglikeness (Lipinski, Violations)	Bioavailability Score	Water Solubility (LogS)
Fucoxanthin	6.78	Low	No	No	No; 2 violations	0.17	−8.20 (Poorly soluble)
Siphonaxanthin	6.53	Low	No	No	No; 2 violations	0.17	−8.00 (Poorly soluble)

BBB: Blood–brain barrier; CYP: Cytochrome P450; GI: Gastrointestinal tract.

## Data Availability

The data presented in this study are available on request from the corresponding author.

## Abbreviations

FX: fucoxanthin; SX: siphonaxanthin; COV-PS02: SARS-CoV-2 pseudovirus; RBD: SARS-CoV-2 chimeric receptor-binding domain; ACE2: angiotensin-converting enzyme 2; GUSAR: General Unrestricted Structure‒Activity Relationships; ADMET: absorption, distribution, metabolism, elimination, and toxicity.
